# Tubular B7-1 expression parallels proteinuria levels, but not clinical outcomes in adult minimal change disease patients

**DOI:** 10.1038/srep41859

**Published:** 2017-02-02

**Authors:** Sung Woo Lee, Seon Ha Baek, Jin Ho Paik, Sejoong Kim, Ki Young Na, Dong-Wan Chae, Ho Jun Chin

**Affiliations:** 1 Department of Internal Medicine, Seoul National University Postgraduate School, Seoul, Republic of Korea; 2 Department of Internal Medicine, Eulji General Hospital, Seoul, Republic of Korea; 3 Department of Internal Medicine, Seoul National University Bundang Hospital, Seongnam, Republic of Korea; 4 Department of Pathology, Seoul National University Bundang Hospital, Seongnam, Republic of Korea

## Abstract

B7-1 is thought to play a pathogenic role in minimal-change disease (MCD). Recently, however, doubts have arisen regarding the role of B7-1 expression in MCD. Therefore, we aimed to identify the presence and clinical significance of B7-1 expression in MCD patients. The study participants included 28 adult MCD patients for whom kidney specimens were available. The intensity of B7-1 expression was assessed by two independent specialists. We analysed the association between the intensity of B7-1 expression and clinicopathological variables. No B7-1 expression in the glomeruli was observed in any of the 28 patients. Unexpectedly, however, 75.0% of the patients exhibited tubular B7-1 expression, with 35.7% demonstrating weak positive expressions and 39.3% demonstrating strong positive expressions. The level of proteinuria significantly increased as the intensity of tubular B7-1 expression increased. We also found trends of increasing blood urea nitrogen and serum creatinine levels with increased intensity of tubular B7-1 expression. However, we could not observe definite differences in long- and short-term clinical outcomes depending on the intensity of tubular B7-1 expression. In conclusion, B7-1 was expressed in renal tubular cells but not in glomeruli in adult MCD patients. The intensity of tubular B7-1 expression paralleled proteinuria levels, but not clinical outcomes.

Minimal-change disease (MCD) accounts for approximately 30% of cases of adult primary nephrotic syndrome (NS)^1^,^2^,^3^. Although MCD is well known for its good response to immunosuppressive drugs^4^,^5^, clinicians also need to keep in mind the high relapse rate^4^,^5^, which causes considerable treatment-related complications^6^. To date, the exact pathogenesis of MCD remains unknown, but T-cell dysfunction is suggested as a central player^7^,^8^,^9^.

B7-1, also known as CD80, is a surface glycoprotein expressed in antigen-presenting cells. As a co-stimulatory signal for T-cell regulation, B7-1 activates T cells by binding to CD 28 or suppresses T cells by binding to CTLA-4^10^,^11^. Since Reiser *et al*. reported an unanticipated role of B7-1 in podocytes as an inducible modifier of glomerular permeability in proteinuric diseases^12^, several studies have been performed to examine the clinical significance of B7-1 expression in patients with MCD^13^,^14^,^15^,^16^,^17^.

However, doubts have arisen regarding the possible role of B7-1 in MCD. First, glomerular B7-1 expression might not be specific for MCD, as it is also found in other types of glomerulonephritis^12^,^18^,^19^. Second, glomerular B7-1 expression might not be a key player in MCD, as B7-1 expression is not always positive^18^ and is even completely negative in some studies^20^,^21^. Finally, B7-1 expression might be induced not only in the glomeruli but also in the tubules^22^,^23^,^24^. To elucidate these uncertainties, we performed the present study in adult patients with biopsy-proven MCD.

## Results

Of the 28 patients, the median age was 49.0 years (range, 25.0–67.0 years) and 57.1% were men. During the follow-up period of 4.4 years (2.8–8.3 years), steroid was prescribed to every patient with a cumulative dose of 11.0 g (5.6–21.1 g) for 14.8 months (5.7–26.1 months). Calcineurin inhibitors and cyclophosphamide were prescribed to 46.4% and 10.7% of patients, respectively. Complete remission (CR) as the maximum clinical response was achieved in 96.4% of the patients, but 50.0% experienced at least one relapse. Two patients (7.1%) died of a stroke and an unidentified reason, respectively. Although none of the patients had a progression to ESRD, one patient developed doubling of creatinine level.

On light microscopic examination, 46.4% of the patients showed glomerular lesions as follows: 17.9%, global sclerosis of ≥10%; 7.1%, glomerular ischemia; 10.7%, increased glomerular cellularity; and 17.9%, increased glomerular size. In addition, 71.4% of the patients showed tubulointerstitial lesions as follows: 64.3%, tubular atrophy; 57.1%, interstitial inflammation; and 60.7%, interstitial fibrosis. On immunofluorescence (IF) examination, the positivity of IgG, IgA, IgM, C3, and C1q were 3.6%, 3.6%, 14.3%, 0%, and 7.1%, respectively.

**Figure 1:**
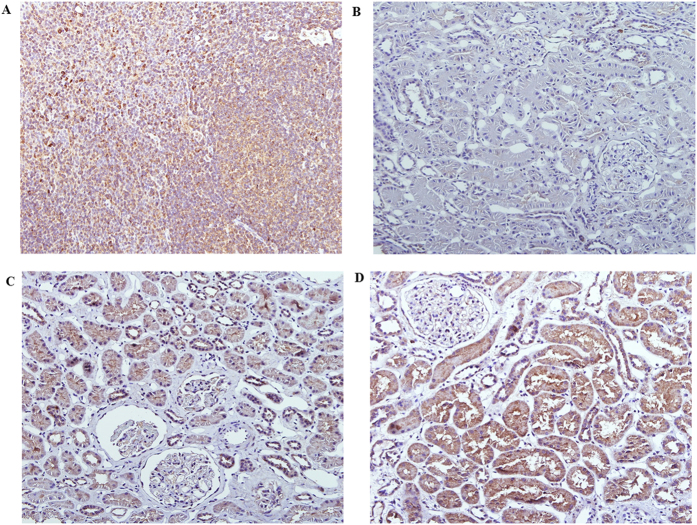
Representative examples of B7-1 expression. (**A**) Strong expression in tonsillar lymphocytes. (**B**) Negative staining in renal tubular epithelial cells and glomeruli. (**C**) Weak expression in renal tubular epithelial cells. (**D**) Strong expression in renal tubular epithelial cells (original magnification, ×200).

**Figure 2:**
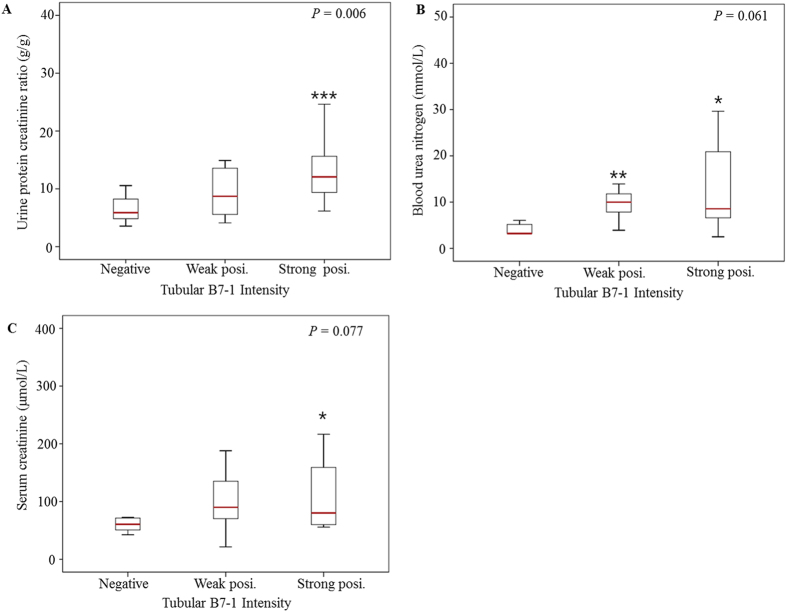
Kidney function and proteinuria according to the intensity of tubular B7-1 expression. (**A**) Urinary protein-to-creatinine ratio. (**B**) Blood urea nitrogen. (**C**) Serum creatinine. Differences were analysed by using the Kruskal-Wallis test. *P < 0.1, **P = 0.033, and ***P = 0.004 by Mann-Whitney *U* test, compared with the tubular B7-1 expression-negative group.

**Figure 3:**
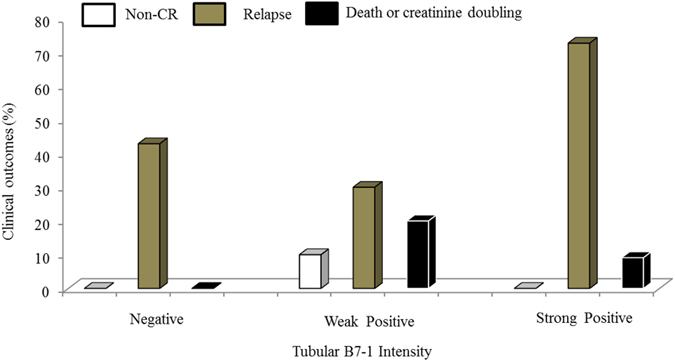
Intensity of the tubular B7-1 expression and clinical outcomes. CR, complete remission. Differences were analysed by using the chi-square test, and all clinical outcomes were not statistically significantly different in terms of the intensity of tubular B7-1 expression.

None of the patients showed B7-1-positive staining results in the glomeruli. However, 75.0% of the patients showed B7-1-positive staining results in renal tubular cells as follows: 35.7% demonstrated weak positive expression and 39.3% demonstrated strong positive expression (Fig. 1). We compared baseline characteristics according to the intensity of tubular B7-1 expression. The level of proteinuria significantly increased with the progression of the intensity of tubular B7-1 expression (Fig. 2A). We also found trends of increasing blood urea nitrogen and serum creatinine levels, with the progression of tubular B7-1 staining intensity (Fig. 2B,C). In parallel with the increased disease severity, more patients were prescribed calcineurin inhibitors with the progression of the intensity of tubular B7-1 expression (Table 1). However, we could not observe statistically significant differences in other clinical, pathological, and therapeutic parameters. In the comparison of clinical outcomes, the intensity of tubular B7-1 expression was not associated with non-CR, relapse, and death, nor with doubling of creatinine level (Fig. 3).

## Discussion

MCD is thought to be a benign disease with excellent long-term prognosis^4^. However, many patients experience relapse^5^, and prolonged immunosuppression could lead to considerable treatment-related complications^6^. Recently, B7-1 expression has been suggested to play a key role in proteinuric renal disease by increasing glomerular permeability^12^, and the activity of B7-1 could be suppressed by CTLA-4 fusion proteins such as abatacept^18^. Therefore, clinicians expect that patients with MCD resistant to the classic treatment might be treated effectively with CTLA-4 fusion protein in cases of positive B7-1 expression. Although several studies have reported the clinical significance of B7-1 expression in MCD^13^,^14^,^15^,^16^,^17^, human studies with adult patients with MCD are lacking. In the present study, we found that B7-1 was not expressed in the glomeruli, but was expressed in renal tubules to varying degrees. This expression was paralleled by proteinuria levels.

Previous studies that described B7-1 expressions in MCD are summarised in Table 2. Among the study groups, one has consistently reported that the urinary B7-1 levels seemed to be higher in patients with MCD in relapse than in those with MCD in remission or other glomerular diseases^13^,^14^,^25^,^26^. To investigate the source of urinary B7-1, they evaluated kidney tissues. They found that kidney tissues from patients with MCD who were in relapse exhibited glomerular B7-1 expression, while tissues from those with MCD in remission or other glomerular diseases did not^14^. However, the findings of these studies cannot be generalised because they only included children for analysis and the number of kidney specimens evaluated was not sufficient (only 8 patients with MCD). Yu *et al*. investigated B7-1 expression in adult patients with various kidney diseases^18^. In the analysis, 5 patients with MCD were included, 60% of whom exhibited glomerular B7-1 expression. On the other hand, Larsen *et al*. reported different results after evaluating B7-1 expression in various proteinuric kidney diseases^20^. Among the 19 adult patients with MCD included, none exhibited glomerular B7-1 expression. This finding was confirmed by the present study and the study by Novelli *et al*.^27^. The main cause of the discrepant results on glomerular B7-1 expression level can be assumed to be the difficult work process involving anti-B7-1 antibodies, particularly in using the IF method to maintain reproducibility and accuracy^20^,^28^. As shown in Table 2, studies with positive glomerular B7-1 expression^14^,^18^ used the IF method only, whereas those with negative glomerular B7-1 expression^20^,^27^ used the immunoperoxidase (IP) method, which is known as not influenced by non-specific binding of secondary antibodies^20^, in addition to the IF method to confirm the results. Therefore, in future studies regarding B7-1 expression in MCD, the IF and IP methods are highly recommended for detection of kidney B7-1 expression.

In the present study, we observed B7-1 expression in renal tubular cells in adult patients with MCD. The results were mostly concordant with those of the previous study by Novelli *et al*.^27^. In their analysis with 15 patients with MCD (age range, 1.7–67.4 years), B7-1 expression was observed in injured epithelial tubular cells. However, they did not analyse the further clinical significance of tubular B7-1 expression. Compared with the above-mentioned research, our study indicates that the level of proteinuria increased with the progression of the intensity of the tubular B7-1 expression. Blood urea nitrogen and serum creatinine levels also showed increasing trends with the progression of the intensity of tubular B7-1 expression, although the statistical significance was marginal. Considering that B7-1 expression correlates with loss of kidney function, an underlying tubular injury may account for the tubular B7-1 expression. In this study, more patients with positive tubular B7-1 expression tended to have tubular atrophy than those with negative tubular B7-1 expression, although the difference was not statistically significant. With these results, we hypothesised that B7-1 may not have a pathogenic role in the development of MCD, but may have an important role in the progression of MCD. Although the clinical outcomes in this study were not affected by the intensity of the tubular B7-1 expression, we assume that the effect of tubular B7-1 expression on MCD progression can be masked by the more-intense treatment administered, with increasing intensity of tubular B7-1 expression. Theoretically, tubular B7-1 expression can potentiate kidney damages by allowing tubules to act as non-professional antigen-presenting cells^23^,^24^,^29^,^30^. Therefore, efforts to determine the role of tubular B7-1 in MCD progression are needed in future experimental or large clinical studies.

The present study has several limitations. First, no stored urine specimens were available for the measurement of B7-1 levels, as this was a retrospective study. Therefore, we cannot provide data regarding urinary B7-1 levels, although it is a good mechanistic connection between tubular B7-1 expression and proteinuria, and a fine non-invasive surrogate biomarker^13^,^14^,^25^,^26^,^31^. Second, selection bias existed because we only included patients for whom biopsy specimens were available. However, this weakness is inevitable in retrospective studies^13^,^14^,^18^,^20^,^22^,^23^. Moreover, the sample size was relatively larger than those of previous studies that examined the significance of tissue B7-1 expression in MCD patients^14^,^18^,^20^,^27^. Third, the intensity of B7-1 expression was assessed semi-quantitatively and subjectively. However, we think that the blinded evaluation by the two independent specialists enhanced the reliability of the assessment of B7-1 expression. Fourth, we used a single B7-1 antibody to detect B7-1 expression. In previous studies^20^,^27^, the IP method was adopted to confirm results from the IF method. The antibody used in this study was for the IP method and was identical to that used in previous studies^20^,^27^. Therefore, we think that the effect of this limitation on the study outcome is acceptable. Finally, as this was a single-centre study, the generalisability of its results is limited.

In conclusion, B7-1 was expressed in renal tubular cells but not in the glomeruli of the adult patients with MCD. The intensity of the tubular B7-1 expression paralleled proteinuria levels but not clinical outcomes. Further studies are needed to elucidate the exact significance of tubular B7-1 expression in adult patients with MCD.

## Methods

### Patients

From 2003 to 2013, 83 patients were diagnosed as having a biopsy-proven MCD at Seoul National University Bundang Hospital (SNUBH), a tertiary care hospital. Among these patients, 70 met the study inclusion criteria as adults with disease classified as MCD with nephrotic-range proteinuria. Four patients aged <15 years, 8 patients with urine protein-to-creatinine ratio (UPCR) of <3.0 g/g, and 1 patient with focal segmental glomerulosclerosis on subsequent biopsy were excluded. After further exclusion of 42 patients for whom kidney specimens were unavailable, we ultimately included 28 patients in the study. We concluded that all the 28 patients had primary MCD because of the lack of evidence of secondary causes such as lupus, IgA nephropathy, active cancer, or current use of non-steroidal anti-inflammatory drugs. This study was approved by the institutional review board (IRB) of SNUBH (IRB No. B-1510/320-114). All the following methods were performed in accordance with the guidelines and regulations of the IRB of SNUBH. The need for informed consent was waived because the study did not infringe on the patients’ privacy or health status.

### Definitions and measurements

Demographic, physiological, laboratory, and therapeutic data were obtained from the electronic medical records database. After different patient datasets were merged, data verification was performed manually. The first date of nephrotic range proteinuria was the start of our study, and the end of the study was a later date between the date of creatinine doubling and onset of end-stage renal disease (ESRD), or the time of death. Definitions of MCD courses are summarised in Supplemental Table 1. Body mass index was calculated as weight (kg) per square of height (m^2^). Serum creatinine level was measured by using the rate-blanked compensated kinetic alkaline picrate Jaffe method with an automatic analyser (Toshiba-200FR, Tokyo, Japan). The between-day coefficients of variation for serum creatinine level were 1.5–2.6% and 1.1–2.4% at low (168.0–176.8 μmol/L) and high concentrations (583.4–627.6 μmol/L), respectively, throughout the study period. Microscopic haematuria was defined as ≥5 red blood cells/high-power field on urine sediment microscopy. Acute kidney injury was defined as an increase in serum creatinine level of ≥0.3 mg/dL within 48 hours or ≥1.5 times higher than that at baseline^32^.

### Kidney pathology

All the specimens were embedded in paraffin and stained with periodic acid-Schiff, Masson trichrome, methenamine silver, and haematoxylin-eosin. Glomerular lesions were defined as global sclerosis of ≥10.0%^33^, increased glomerular size or cellularity, or the presence of glomerular ischemia. Tubular atrophy, and interstitial inflammation and fibrosis were defined as non-normal reports^34^. Vascular lesions were defined as the presence of arteriolar hyalinosis and arteriosclerosis. Detailed information regarding the IF study methods are described elsewhere^4^. In brief, the classic direct technique with antibodies against 5 antigens (immunoglobulin [Ig] G, IgM, IgA, C3, and C1q) was used. Positive IF staining was defined as a score of >2 in the sum of linear-, granular-, peripheral-, and mesangial-term in the glomerulus.

### B7-1 immunohistochemical staining

We performed immunohistochemical staining for B7-1 by using a BenchMark XT automated immunostaining system (Ventana Medical Systems, Inc., Tucson, AZ, USA). Briefly, the paraffin blocks were cut into 4-μm-thick sections, deparaffinised in xylene, and then hydrated by using alcohol (3×). After microwave antigen retrieval, the samples were incubated with a monoclonal mouse anti-human B7-1 antibody (diluted 1:20; catalogue No. MAB140; R&D Systems, Minneapolis, MN, USA) and subsequently treated with the UltraView Universal DAB kit (Ventana Medical Systems, Inc.). Harris haematoxylin was used as a counterstain. Two independent specialists blinded to the patients’ clinical data evaluated the stained slides. If the interpretation of the staining results was not consistent, the two specialists discussed conflicting findings. The intensity of the signal was rated 0 (negative), 1 (weak), or 2 (strong). Staining results for the glomerulus and tubular epithelium were recorded separately. Normal tonsillar tissue was used as a positive control for B7-1.

### Statistical analysis

Values were expressed as median (interquartile range) for continuous variables and % (n/total) for categorical variables. The difference was analysed by using the Kruskal-Wallis test or Mann-Whitney *U* test for continuous variables, and the Chi-square or Fisher exact test for categorical variables. A *P* value of <0.05 was considered statistically significant. All the analyses were performed by using SPSS Statistics version 22 (IBM Corp, Armonk, NY, USA).

## Additional Information

**How to cite this article**: Lee, S. W. *et al*. Tubular B7-1 expression parallels proteinuria levels, but not clinical outcomes in adult minimal change disease patients. *Sci. Rep.* **7**, 41859; doi: 10.1038/srep41859 (2017).

**Publisher's note:** Springer Nature remains neutral with regard to jurisdictional claims in published maps and institutional affiliations.

## Supplementary Material

Supplementary Information

## Figures and Tables

**Table 1:** Baseline characteristics according to the intensity of tubular B7-1 expression.

	Negative (n = 7)	Weak Positive (n = 10)	Strong Positive (n = 11)	*P*
Age (yr)	37.9 (27.4–71.9)	60.5 (30.0–67.2)	27.6 (23.8–67.5)	0.497
Male sex	4 (57.1)	6 (60.0)	6 (54.5)	0.969
SBP (mm Hg)	111.0 (109.0–121.0)	128.0 (114.0–135.0)	114.0 (105.0–132.0)	0.650
DBP (mm Hg)	70.0 (64.0–75.0)	77.0 (60.0–83.0)	76.0 (57.0–78.0)	0.296
BMI (kg/m^2^)	28.1 (22.3–30.6)	24.6 (23.2–26.1)	22.9 (21.3–24)	0.135
Acute kidney injury	2 (28.6)	2 (20.0)	3 (27.3)	0.900
Haemodialysis	0 (0.0)	0 (0.0)	1 (9.1)	0.449
Glucose (mmol/L)	4.9 (4.8–5.3)	5.4 (5.1–6.6)	6.2 (5.1–6.8)	0.094
Protein (g/L)	45.0 (41.0–47.0)	45.0 (41.0–51.0)	44.0 (41.0–45.0)	0.648
Albumin (g/L)	21.0 (18.0–22.0)	24.0 (20.0–27.0)	21.0 (20.0–24.0)	0.437
Cholesterol (mmol/L)	12.6 (7.4–14.0)	10.1 (7.1–13.8)	13.3 (7.4–13.4)	0.650
Fibrinogen (g/L)	7.3 (6.0–9.1)	6.2 (5.8–7.6)	7.1 (5.7–8.1)	0.688
WBC (×10^3^/μL)	5.9 (5.4–7.0)	6.7 (5.3–7.9)	6.0 (5.2–8.1)	0.964
Haemoglobin (g/dL)	15.2 (14.1–16.2)	13.8 (12.2–14.3)	14.8 (12.7–16.7)	0.651
Platelet (×10^3^/μL)	228.0 (190.0–273.0)	267.0 (201.0–293.0)	254.0 (200.0–286.0)	0.650
Microscopic haematuria	1 (14.3)	5 (50.0)	3 (27.3)	0.272
Glomerular lesions	4 (57.1)	3 (30.0)	6 (54.5)	0.428
Tubular atrophy	4 (57.1)	7 (70.0)	7 (63.6)	0.861
Interstitial inflammation	3 (42.9)	6 (60.0)	7 (63.6)	0.668
Interstitial fibrosis	4 (57.1)	6 (60.0)	7 (63.6)	0.961
Vascular lesions	3 (42.9)	4 (40.0)	3 (27.3)	0.749
Steroid duration (days)	559.0 (272.0–949.0)	379.0 (150.0–768.0)	431.0 (180.0–788.0)	0.892
Steroid dosage (g)	11.0 (8.9–32.5)	8.3 (5.3–23.6)	11.6 (3.5–16.1)	0.684
CNI use	1 (14.3)	4 (40.0)	8 (72.7)*	0.047
Cyclophosphamide use	2 (28.6)	1 (10.0)	0 (0.0)	0.161

SBP, systolic blood pressure; DBP, diastolic blood pressure; BMI, body mass index; WBC, white blood cells; CNI, calcineurin inhibitor.

Values are expressed as median value (interquartile range) for continuous variables and n (%) for categorical variables. Differences were analysed by using the Kruskal-Wallis test for continuous variables and the chi-square test for categorical variables.

*P < 0.05 by Fisher exact test, compared with the tubular B7-1 expression-negative group.

**Table 2:** Previous studies on B7-1 expression in minimal-change disease.

Authors	n*	B7-1 Measurement	B7-1 Expression	Limitations
**Using kidney tissue**				
Garin^14^	8	IF	Glomeruli (87.5%); tubules (0%)	1. No adult patients
				2. No confirmation with IP
				3. No grading for B7-1 expression intensity
Yu^18^	5	IF	Glomeruli (60.0%); tubules (not evaluated)	1. No confirmation with IP
				2. No grading for B7-1 expression intensity
Larsen^20^	19	IF and IP	Glomeruli (0%); tubules (not evaluated)	1. No grading for B7-1 expression intensity
Novelli^27^	15	IF and IP	Glomeruli (0%); tubules (presented)	1. No grading for tubular B7-1 expression intensity
**Using urine**				
Garin^13^	19	ELISA	Higher urinary B7-1 level in MCD relapse than in MCD remission or other glomerular diseases	1. No adult patients
				2. No data regarding source of B7-1
Garin^14^	17	ELISA	Higher urinary B7-1 level in MCD relapse than in MCD remission or FSGS	1. No adult patients
				2. Indirect data regarding source of B7-1
Cara-Fuentes^25^	32	ELISA	Higher urinary B7-1 level in MCD relapse than in MCD remission or healthy controls	1. No adult patients
				2. No data regarding source of B7-1
Cara-Fuentes^26^	26	ELISA	Higher urinary B7-1 level in MCD relapse than in MCD remission, FSGS, or controls	1. No adult patients
				2. No data regarding source of B7-1
Ling^31^	37	ELISA	Higher urinary B7-1 level in MCD than in FSGS, other glomerular diseases, or controls	1. No adult patients
				2. No data regarding source of B7-1

MCD, minimal change disease; FSGS, focal segmental glomerulosclerosis; IF, immunofluorescence; IP, immunoperoxidase; ELISA, enzyme-linked immunosorbent assay.

In all the above-mentioned studies, IF was performed by using polyclonal goat anti-human B7-1 (R&D systems, Minneapolis, MN, USA) and IP was analysed by using monoclonal mouse anti-human B7-1 (R&D systems, Minneapolis, MN, USA). For the measurement of urinary B7-1 level, researchers in all the above-mentioned studies used a commercial ELISA kit (Bender MedSystems, Burlingame, CA, USA). As none of the previous studies graded the intensity of B7-1 expression, quantitatively or semi-quantitatively, no data were available on the association of the intensity of B7-1 expression with clinical outcomes. *Only numbers of specimens from MCD patients were counted.
