# Supplementary material for: Tubular B7-1 expression parallels proteinuria levels, but not clinical outcomes in adult minimal change disease patients
Source: Sci Rep. 2017 Feb 2;7:41859. doi: 10.1038/srep41859 (PMC5288792; doi:10.1038/srep41859)
Supplement: Supplementary Information [file srep41859-s1.pdf]

Tubular B7-1 expression parallels proteinuria levels, but not clinical outcomes in adult minimal change disease patients

Sung Woo Lee[1,2], Seon Ha Baek[3], Jin Ho Paik[4], Sejoong Kim[3], Ki Young Na[1,3], Dong-Wan Chae[1,3], and Ho Jun Chin[3]

[1]*Department of Internal Medicine, Seoul National University Postgraduate School, Seoul, Korea*

[2]*Department of Internal Medicine, Eulji General Hospital, Seoul, Korea*

[3]*Department of Internal Medicine, Seoul National University Bundang Hospital, Seongnam, Korea*

[4]*Department of Pathology, Seoul National University Bundang Hospital, Seongnam, Korea*

Correspondence to:

Ho Jun Chin

e-mail: mednep@snubh.org Tel) 82-31-787-7025 Fax) 82-31-787-4052

Mailing address) Department of Internal Medicine, Seoul National Univ. Bundang Hospital, Gumi-dong 300, Bundang-gu, Seongnam-si, Gyeonggi-do, 463-707, Korea

Supplementary Table 1. Definitions of minimal change disease courses

MCD courses	Definition
Nephrotic range proteinuria	UPCR ≥ 3.0 g/g or ≥ 3+ on urine dipstick for 3 consecutive days
Complete remission	UPCR < 0.3 g/g or < 1+ on urine dipstick for 3 consecutive days
Partial remission	Reduction of UPCR ≥ 50% from the initial level, with < 3.0 g/g absolute value
Relapse	Reappearance of nephrotic range proteinuria after remission
Creatinine doubling	2 times increase in serum creatinine level compared to serum creatinine level at biopsy
Death	Positive record in the database of Korean Statistics.
End stage renal disease	Positive record in the registry database of the Korean Society of Nephrology

UPCR, urine protein-to-creatinine ratio
